# Effectiveness of a blended learning intervention in cardiac physiotherapy. A randomized controlled trial

**DOI:** 10.3389/fpubh.2023.1145892

**Published:** 2023-05-09

**Authors:** Elena Marques-Sule, Juan Luis Sánchez-González, Juan J. Carrasco, Sofía Pérez-Alenda, Trinidad Sentandreu-Mañó, Noemí Moreno-Segura, Natalia Cezón-Serrano, Regina Ruiz de Viñaspre-Hernández, Raúl Juárez-Vela, Elena Muñoz-Gómez

**Affiliations:** ^1^Physiotherapy in Motion, Multispecialty Research Group, Department of Physiotherapy, Faculty of Physiotherapy, University of Valencia, Valencia, Spain; ^2^Department of Nursing and Physiotherapy, University of Salamanca, Salamanca, Spain; ^3^Department of Physiotherapy, Faculty of Physiotherapy, University of Valencia, Valencia, Spain; ^4^Nursing Department, Faculty of Health Sciences, Research Group GRUPAC, University of La Rioja, Logroño, Spain

**Keywords:** physiotherapy, teaching methods, learning, undergraduate (MeSH), education

## Abstract

**Background:**

Blended learning (BL) combines both face-to-face learning (FL) and online learning. This study aims to compare the effectiveness of a BL intervention vs. a FL intervention in relation to the knowledge, competencies, satisfaction, perceptions, usability, and BL acceptance of physiotherapy students.

**Methods:**

An assessor-blinded randomized trial was performed. A total of 100 students were randomly allocated to either the BL group (BLG, *n* = 48) or FL group (FLG, *n* = 52). The BLG received face-to-face classes plus access to online resources (online syllabus, Moodle, scientific-based videos and websites, activities, glossary, and apps). The FLG received face-to-face classes and hardcopy resources (hardcopy syllabus, scientific-based information, activities, and a glossary). Knowledge, ethical and gender competencies, satisfaction, perceptions, usability, and BL acceptance were assessed.

**Results:**

The BLG showed higher scores than the FLG for knowledge (*p* = 0.011), three ethical/gender competencies (*p* < 0.05), increased motivation to prepare themselves before class (*p* = 0.005), increased motivation and ability of thinking (*p* = 0.005), improved understanding of important topics (*p* = 0.015), course organization (*p* = 0.017), educational material (*p* = 0.001), easiness of understanding (*p* = 0.007), comprehensive coverage of the subject (*p* = 0.001), and clarity of instructions (*p* = 0.004), while usability was acceptable.

**Conclusion:**

The BL intervention can be used for improving the knowledge, competencies, perceptions, and satisfaction of the students. In addition, BL acceptance was positive, and usability was found to be acceptable. This study supports the use of BL as a pedagogical approach to foster innovative learning.

## Introduction

The confluence between online learning and face-to-face resources is becoming a reality today (1). Moreover, the coronavirus disease-2019 (COVID-19) pandemic has been a challenge for educational institutions worldwide. In the countries where the governments decided to apply total containment, students were unable to attend their regular classes (2). Thus, the teachers had to move from the traditional face-to-face classroom to online learning, which was a good opportunity and a great challenge for university teaching (3–5).

This transformation was a novel situation for several health science degrees, as well as the physiotherapy degree, where teaching had traditionally been face-to-face learning (FL)-based due to including both theoretical and practical classes (6). Moreover, the unprecedented situation led to the fact that there was not enough time given for extensive training on online teaching skills, and the impossibility of changing the course content. Teachers faced the challenge trying to teach the core competencies of their subjects through online teaching (7, 8). In addition, the physiotherapy students had to cope with the psychosocial issues arising from the pandemic (9, 10) while getting used to the new teaching modalities (6).

In this regard, the pre-pandemic literature, such as the study performed by Unge et al., have criticized the online approach, arguing that the physiotherapy degree is not grounded in a theoretical learning perspective. It was also stated that it seems to be organized based on a short perspective rather than aligned to a whole intervention (11). In contrast, some systematic reviews state that online learning is effective and generates the same satisfaction as traditional FL in physiotherapy, concluding that online learning is equivalent and possibly even superior to traditional learning (12–14). In addition, digital, social, and mobile technologies are increasingly used in health professional education and students have reported high levels of satisfaction following the use of these technologies (15). It should be noted that other studies have emphasized that, although the students are satisfied with e-learning, traditional instructor-led training should not be replaced and instead could be a complement. This is the basis of the blended learning (BL) approach (16, 17).

The interaction between the teacher and students is necessary to learn directly from the knowledge and clinical experience of the teacher as well as to develop real-time group discussions with the students (18). In addition, the face-to-face methodology is an excellent technique for people who require this form of effective communication and a personal connection of their work with their own values, both of which are necessary for healthcare professionals in their dealings with patients (19). However, innovative ways to engage in FL are also needed to improve the quality of the teaching methods. The pre-pandemic studies that compared two types of methodologies such as online learning or BL vs. FL do not propose to substitute one for the other. These studies suggest that new methodologies serve as support in the learning process of the students without losing the benefits of FL (20).

BL alludes to the convergence between two learning environments, the face-to-face environment which has a long tradition in our educational system, and the online environment which expands and modifies the possibilities of communication and interaction (21). Previous studies have showed that BL environments in which a reduction of the classroom time between 30 and 79% was performed were not associated with poorer learning outcomes and were equivalent to conventional classroom instruction (22). BL is revolutionizing distance education, not only with regards to the quality of the teaching-learning process but also in terms of user satisfaction and accessibility (22). This BL approach may offer pedagogical benefits while maintaining an advantageous level of social interaction (23). In this sense, BL has been proven to have a number of advantages. For example, students can prepare themselves prior to the class to improve the effectiveness of the learning activities or even after class in order to assimilate the acquired knowledge. This learning modality prepares the students for the new challenge of facing the daily use of technology in the context of physiotherapy and other healthcare sciences as part of the development of the profession. Furthermore, BL eliminates the barriers of time and distance and increases cost-effectivity (24–26). In addition, this learning model also gives more prominence to the student since it moves from an educator-centered teaching model to a context where the student becomes more active, carrying out processes that expand their knowledge, as well as the transformation of the role of the teacher from a knowledge diffuser to a facilitator (17, 26).

Before the outbreak of the COVID-19 pandemic, BL was already recommended due to using active learning strategies that enriched the learning experience. Many investigations have been developed to conceptualize the model, explain the different methods and/or reporting their benefits (21) but post-COVID-19 and an increase in the literature has since occurred (2, 21). Numerous studies have proven the effectiveness of BL in different healthcare disciplines (17, 27–29) and have explored student satisfaction in this regard. For example, Güzer and Caner (26) determined that BL is useful, supportive, flexible and motivating, and reported greater results than FL in terms of satisfaction, motivation, dropout rate, attitudes, retention of information, etc. More recently, Kang and Kim showed that BL instructional methods, compared with traditional lectures, enhanced the students' knowledge, problem-solving ability, and learning satisfaction in a public healthcare course (30). The systematic review and metanalysis carried out by Du et al. found that BL may be an effective teaching strategy in nursing education and that it appears to have excellent long-term developmental potential. They emphasize that the initial construction required a specific investment to improve the teaching resources and standardize the design of BL in the long term (31). However, several systematic reviews emphasize the importance of pursuing further studies that analyze the effect of this alternative approach in relation to each of the disciplines in which this method could be implemented (12, 22, 26). Further research is needed to determine the usefulness of this teaching modality in different disciplines to minimize the difficulties encountered in its implementation and to provide new strategies and environments in which its educational effectiveness may be demonstrated (24).

Therefore, this study aimed to determine the effectiveness of a BL intervention vs. a FL intervention on knowledge, cross-curricular competencies, satisfaction, perceptions, usability, and BL acceptance among physiotherapy degree students. We hypothesized that the students performing the BL intervention would show better results than those performing the FL intervention.

## Materials and methods

### Participants and study design

Simple sampling was performed. Undergraduate physiotherapy students were recruited from the University of Valencia (Spain) in September 2020. The inclusion criteria were those studying the subject of cardiac physiotherapy as part of the Degree of Physiotherapy at the University of Valencia and those who were willing to participate. Students with previous cardiac physiotherapy training were excluded.

This was a longitudinal single-blinded randomized trial with two arms of intervention (registration with ClinicalTrials.gov number NCT05547009). The trial was conducted following the CONSORT extension for pragmatic clinical trials (32). The participants were randomly allocated to two groups, either the BL group (BLG) or the FL group (FLG). Both interventions lasted 2 months and included five face-to-face lessons and autonomous work using different methodologies according to the assigned group. The participants were assessed at baseline and after the intervention.

### Randomization and masking

Randomization was undertaken by an independent research assistant not involved in the trial who prepared a computer-generated random allocation sequence. Group allocation was revealed to the study members once the participants had completed all baseline procedures. The outcome assessor was blinded to the group allocation data. The blinded assessor collected all baseline and post-intervention measures and entered the data.

### Outcome measures

All participants provided demographic information, including age, gender, marital status, if they had any other university degrees, occupational status, and how many hours they were working per week. Assessments were conducted by a teacher with more than 10 years of experience in cardiac physiotherapy. The following outcomes were assessed:

a) Knowledge acquisition was measured using a multiple-choice test (33). Students were not previously informed about the retention exam to avoid any preparation for the test. The tests included 10 multiple-choice questions that assessed their knowledge of cardiac physiotherapy-related issues including topics such as the phases of cardiac rehabilitation; warm-up, exertion, and cool-down; physical activity and exercise assessment; heart rate and exercise; and frequency, intensity, duration, and type of exercise.
b) Cross-curricular ethical and gender competencies were measured using the Higher Education, Transversal Skills and Gender Questionnaire (34). The tool was composed of six items using a 7-point Likert scale (from 1 = totally disagree to 7 = totally agree). The maximum questionnaire score was 42 points. The higher the score, the more ethical and gender competencies were acquired. This questionnaire has shown a high reliability coefficient (87 %) (34).
c) Satisfaction was assessed using a four-point Likert-scale (1 = poor, 4 = excellent) using the scale utilized by Yang et al. (35). This scale measured aspects such as motivation, activating class atmosphere, teamwork, clinical problem-solving skills, and the understanding of difficult topics. The higher the score, the better the satisfaction.
d) The perceptions and evaluation of the intervention were evaluated using the 8-item Kavadella Perceptions Questionnaire (36) that was scored on a five-point Likert scale. It evaluated the students' perceptions about the course content, course organization, educational material, easiness to understand, comprehensive coverage of the subject, online course design, clarity of instructions, motivating study timetable, and self-assessment tests. The higher the score, the better the perceptions and evaluation of the intervention.
e) The usability of the Moodle Virtual Classroom platform was measured using the System Usability Scale (SUS) (37, 38). The SUS includes 10 items covering three facets of usability: effectiveness, efficiency, and satisfaction. It was scored on a 5-point Likert scale from 1 (strongly disagree) to 5 (strongly agree). The total score was calculated in two steps that entailed transformation and reversion to a 4-point scale, and the calculation of the total score by summing the scores of all 10 items and multiplying by 2.5. The total SUS score (0–100) can be interpreted as not acceptable (0–64), acceptable (65–84), or excellent (85–100) (39, 40). The internal consistency of the SUS was found to be high (α = 0.91), and the concurrent validity was found to be moderate (*r* = 0.81, *p* < 0.001) (41). The higher the score, the better the usability of the platform. BL acceptance was evaluated using the 4-item Blended Learning Acceptance Scale (42) which represents four constructs in the Technology Acceptance Model (perceived ease of use, perceived usefulness, attitude toward use, and behavioral intention). These items were drawn from a previous study on the acceptance of BL (43). The items were scored on a Likert scale ranging from 1 (never true) to 7 (always true) (42). This questionnaire showed values >0.7 (ranging between 0.786 and 0.927) which implies a good composite reliability and rho with a resulting measure between 0.712 and 0.917 (44). The higher the score, the better the acceptance of the intervention.

Ethical and gender competencies, student satisfaction, and perceptions were measured at baseline and after completing the study (2 months) for both groups, while knowledge acquisition was measured at the end of the study. Additionally, usability and BL acceptance were assessed at the end of the study in the BLG.

### Intervention

A physiotherapy teacher with over 10 years of experience in cardiac physiotherapy performed the teaching methodology. Both groups followed a 2-month intervention. The content of the information provided was the same for both groups. The content and training for both groups was identical, and the only aspect that varied was the methodology.

The interventions were as follows.

a) Face-to-face learning group:

The FLG received the same content as the BL group in terms of the scheduled face-to-face classes and material for further knowledge. However, all information was provided on paper. The FL intervention included synchronous scheduled face-to-face lectures (five in total) and scheduled activities offered at set points during the semester. The face-to-face resources used were as follows: (1) A hardcopy specific syllabus about cardiac physiotherapy that consisted of five themes that included topics as epidemiology and primary prevention, clinical and complementary assessments, phases I, II, and III, cardiac physiotherapy in heart failure, and cardiac physiotherapy in heart transplantation; (2) Hardcopy glossary comprised of terms such as primary prevention, secondary prevention, atherosclerosis, coronary artery bypass grafting, percutaneous coronary intervention, and percutaneous coronary intervention; (3) Hardcopy information by international and national scientific societies was provided which included information regarding heart disease, as well as reinforcing the concepts of professional deontology, equity, clinical status, and the experiences of vulnerable patients with heart disease; (4) Five hardcopy four-question activities were created. Personalized comments about the activities were reported by the teachers to give feedback to the students; and (5) Face-to-face tutorship.

b) Blended-learning group:

The BL intervention included paced asynchronous online learning modules and scheduled online activities with strategic face-to-face lectures offered at set points during the semester. The students had autonomy and flexibility when accessing the online course content except for the intermittently scheduled face-to-face classes (five in total). The online resources used were as follows: (1) Moodle platform where the students were able to read breaking news, have access to the teaching guide, and solve any doubt through discussion forums; (2) Online syllabus about cardiac physiotherapy consisting of five themes, including the same topics as the FLG; (3) Online glossary comprised of the same terms as the glossary developed for the BLG; (4) Online videos by international and national scientific societies, also aimed at improving the same aspects as reported in the FLG, such as knowledge regarding heart disease as well as reinforcing the concepts of professional deontology, equity, clinical status, and the experiences of vulnerable patients with heart disease; (5) Several links to the websites of international and national scientific societies such as the European Society of Cardiology, the American Heart Association, and the World Heart Federation; (6) Online activities with the same four questions per theme as the FLG were created, and personalized comments about the activities were also reported by the teachers in order to give feedback to the students; (7) the app Ariadna (Spanish Society of Cardiology) used to prevent cardiovascular risk and to look for defibrillators in the area; and (8) Online tutorship through an online communication platform (Blackboard Collaborate). This is a simple and reliable virtual classroom solution to power online teaching needs (45). In all cases, the content of the information provided was the same as the information provided to the FLG.

### Statistical analysis

Data analysis was performed using the IBM SPSS Statistics software (Version 22.0; IBM Corp, Armonk, NY, USA). The Kolmogorov-Smirnov test was used to verify the normality of the data. The descriptive data are shown using the mean (standard deviation), median (25–75th percentile), and absolute frequency (percentage) as appropriate. Inferential analysis was performed in order to identify the differences between the groups (BLG and FLG) through the Wilcoxon rank sum test for the continuous variables and Chi-square test (χ^2^) for the categorical ones. The pre-post differences were assessed using the Wilcoxon signed rank test. The effect size was calculated according to the Wilcoxon *z*-values. The eta squared values obtained were then converted to *d*-Cohen values and interpreted as small (*d* = 0.2), medium (*d* = 0.5), and large (*d* > 0.8). Statistical significance was set at *p* < 0.05.

## Results

**Figure 1:**
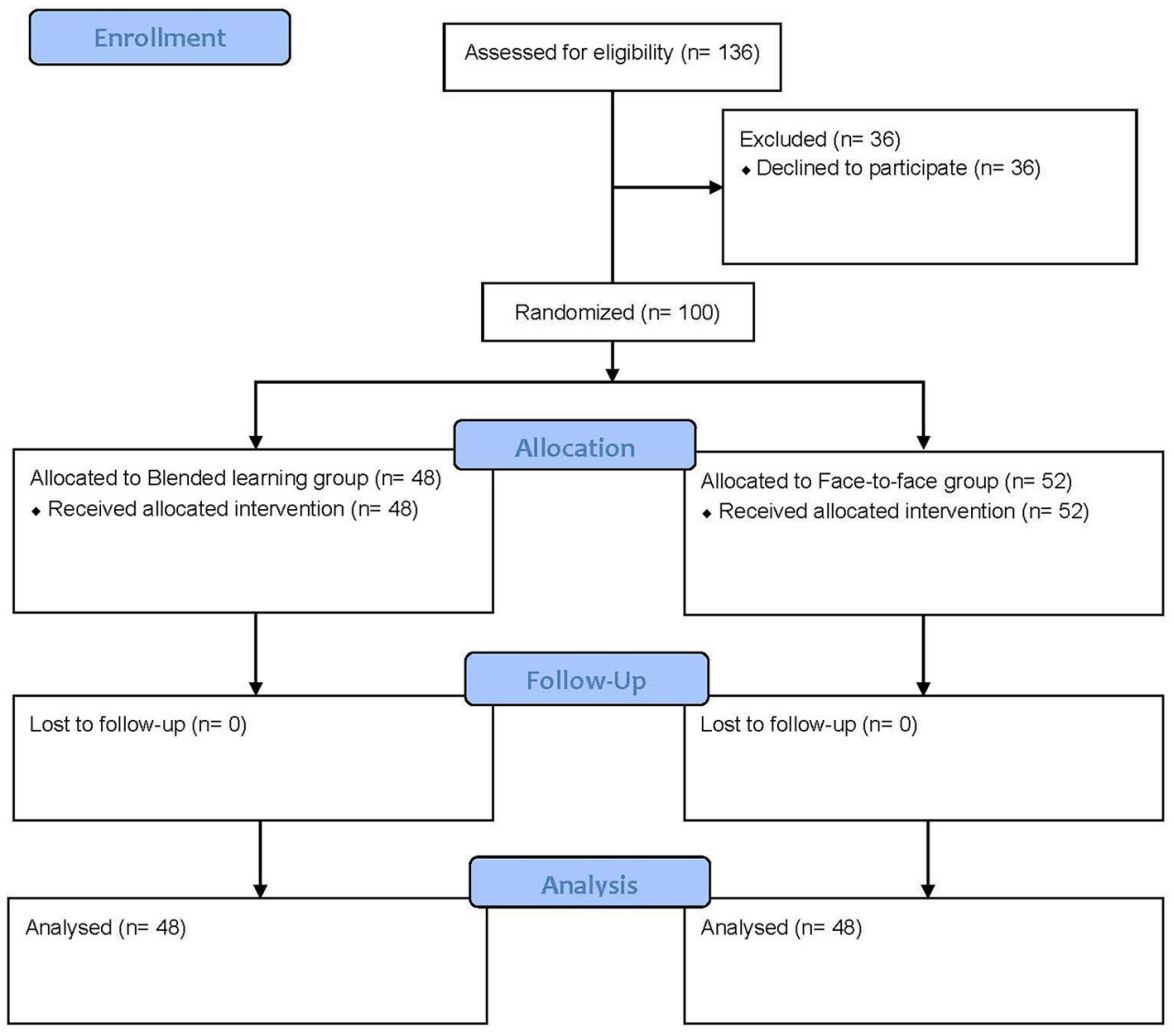
CONSORT flow diagram of the progress through the phases of the study.

From a total of 136 students who fulfilled the inclusion criteria, 100 of them (aged 19–31 years) took part in the study (BLG *n* = 48, FLG *n* = 52). The main reason for exclusion was unwillingness to participate (*n* = 36). Figure 1 shows the flow chart of the study according to the CONSORT statement for the reporting of randomized trials. Table 1 summarizes the students' demographic variables. There were no significant differences between the groups (*p* > 0.05).

**Table 1:** Baseline subject characteristics.

	**BLG (*n* = 48)**	**FLG (*n* = 52)**	
Age (years)	21.1 ± 2.0	20.7 ± 1.8	*Z* = −0.956;
	20.0 (20.0–21.8)	20.0 (20.0–21.0)	*p* = 0.339
**Gender**			χ^2^ (1) = 0.000;
Male	23 (47.9%)	25 (48.1%)	*p* = 0.987
Female	25 (52.1%)	27 (51.9%)	
**Marital status**			χ^2^ (1) = 0.932;
Single	48 (100.0%)	51 (98.1%)	*p* = 0.334
Married	0 (0.0%)	1 (1.9%)	
**Other university degree**			χ^2^ (1) = 0.003;
Yes	1 (2.1%)	1 (1.9%)	*p* = 0.954
No	47 (97.9%)	51 (98.1%)	
**Occupation status**			χ^2^ (2) = 0.014;
Unemployed	44 (91.6%)	48 (92.3%)	*p* = 0.993
Work < 10 h a week	3 (6.3%)	3 (5.8%)	
Work 10–20 h a week	1 (2.1%)	1 (1.9%)	

Data are expressed as mean ± standard deviation and median (25–75th percentile) or absolute frequency (%). BLG, blended learning group; FLG, face-to-face learning group.

Table 2 shows the results of the pre-post and between-group comparison analysis used to assess the ethical and gender competencies questionnaire. After the intervention, there were no significant differences compared to the baseline in the BLG. However, a statically significant score reduction was found in the FLG for item 5 (“Be able to project the knowledge, skills and abilities acquired to promote a society based on the values of freedom, justice, equality and pluralism”) (*p* < 0.001; *d* = 1.060) and in the total score (*p* = 0.023; *d* = 0.664). Furthermore, the BLG presented with significantly higher scores than the FLG for item 4 (“I consider equality an important value in the development of my future”) (*p* = 0.014; *d* = 0.721), item 5 (*p* = 0.001; *d* = 1.095) and item 6 (“Be able to develop skills related to smart development of projects using various conceptual and practical tools, as well as the ability to use them creatively attending to criteria of equity and professional deontology”) (*p* = 0.001; *d* = 1.021). Nevertheless, there were no significant differences between the groups in the total score of the questionnaire (*p* > 0.05).

**Table 2:** Results for the ethical and gender competencies before and after the intervention in both groups.

	**BLG (*****n*** = **47)**		**FLG (*****n*** = **52)**		**Post-intervention differences between groups**
	**Pre**	**Post**	**Pre**	**Post**	
	**Mean** ±**SD**	**Mean** ±**SD**	**Mean** ±**SD**	**Mean** ±**SD**	
	**Median (IQR)**	**Median (IQR)**	**Median (IQR)**	**Median (IQR)**	
**Cross-curricular ethical and gender competencies (1–7 points)**					
1—I believe that well-done collaborative work surpasses individualistic and competitive work related to gender equality	6.1 ± 1.4	6.3 ± 1.1	6.3 ± 1.1	6.2 ± 1.0	*Z* = −0.553
	7.0 (5.0–7.0)	7.0 (6.0–7.0)	7.0 (6.0–70)	6.5 (6.0–7.0)	*p* = 0.580 *d* = 0.154
2—I consider it necessary to maintain an ethical commitment in the development of my professional work related to gender equality	7.0 ± 0.2	6.7 ± 1.1	6.8 ± 0.4	6.7 ± 0.6	*Z* = −1.084
	7.0 (7.0–7.0)	7.0 (7.0–7.0)	7.0 (7.0–7.0)	7.0 (6.0–7.0)	*p* = 0.278 *d* = 0.304
3—I consider it essential to address professional issues of an ethical nature based on gender issues in the different subjects of the degree	6.2 ± 1.1	6.2 ± 1.2	6.4 ± 0.8	6.3 ± 0.9	*Z* = −0.103
	7.0 (6.0–7.0)	7.0 (6.0–7.0)	7.0 (6.0–70)	7.0 (6.0–7.0)	*p* = 0.918 *d* = 0.029
4—I consider equality to be an important value in the development of my future profession	6.8 ± 0.6	6.8 ± 1.0	6.6 ± 0.8	6.5 ± 0.9	*Z* = −2.447
	7.0 (7.0–7.0)	7.0 (7.0–7.0)	7.0 (6.0–70)	7.0 (6.0–7.0)	*p* = 0.014^*^d = 0.721
5—Be able to project the knowledge, skills, and abilities acquired to promote a society based on the values of freedom, justice, equality, and pluralism	7.0 ± 0.2	6.8 ± 1.0	6.8 ± 0.5	6.4 ± 0.8	*Z* = −3.462
	7.0 (7.0–7.0)	7.0 (7.0–7.0)	7.0 (7.0–7.0)	7.0 (6.0–7.0)^**^	*p* = 0.001^*^d =1.095
6—Be able to develop skills related to the smart development of projects using various conceptual and practical tools, as well as the ability to use them creatively attending to the criteria of equity and professional deontology	6.7 ± 0.5	6.7 ± 1.0	6.5 ± 0.6	6.3 ± 0.8	*Z* = −3.28
	7.0 (6.0–7.0)	7.0 (7.0–7.0)	7.0 (6.0–70)	6.0 (6.0–7.0)	*p* = 0.001^*^d =1.021
Total score	39.8 ± 2.2	39.4 ± 5.1	39.3 ± 3.1	38.4 ± 3.9	*Z* = −1.552
	40.0 (39.0–41.5)	41.0 (39.0–42.0)	40.0 (37.3–42.0)	40.0 (35.3–42.0)^**^	*p* = 0.121 *d* =0.441

Data are expressed as mean **±** standard deviation and median (interquartile range). BLG, blended-learning group; FLG, face-to-face learning group. ^*^Significant differences between groups. ^**^Pre-post significant differences.

Regarding knowledge acquisition (Table 3), the BLG showed a significantly higher score than the FLG group (*p* = 0.011; *d* = 0.827) after the interventions. In relation to satisfaction with the methodology, the BLG showed higher scores for increased motivation to prepare themselves before class (*p* = 0.005; *d* = 0.974), increased motivation and ability of thinking (*p* = 0.005; *d* = 0.987), and improved understanding of important and difficult topics (*p* = 0.015; *d* = 0.822) than the FLG. However, there were no significant differences between the groups for active class atmosphere, promoted teamwork spirit and ability, or developed clinical problem-solving skills (*p* > 0.05). Finally, regarding the perceptions and evaluation of the intervention, the BLG presented with higher scores for course organization (*p* = 0.017; *d* = 0.828), educational material (*p* = 0.001; *d* = 1.236), easiness to understand (*p* = 0.007; *d* = 0.958), comprehensive coverage of the subject (*p* = 0.001; *d* = 1.218), and clarity of instructions (*p* = 0.004; *d* = 1.026) compared to the FLG. In contrast, there were no significant differences between the groups in terms of perception and evaluation regarding course content, motivating study timetable, and the self-assessment test (*p* > 0.05).

**Table 3:** Results for knowledge acquisition, satisfaction, and perceptions after the intervention.

	**BLG (*****n*** = **47)**		**FLG (*****n*** = **52)**		
	**Mean** ±**SD**	**Median (IQR)**	**Mean** ±**SD**	**Median (IQR)**	**Post-intervention differences between groups**
**Knowledge acquisition (1**−**10) points**					
	7.2 ± 1.3	7.0 (6.0–8.0)	6.3 ± 1.6	7.0 (5.5–7.0)	*Z* = −2.535 *p* = 0.011^*^d = 0.827
**Satisfaction with the methodology (1–4 points)**					
Increased motivation to prepare before class	2.0 ± 1.0	2.0 (1.0–3.0)	1.5 ± 0.6	1.0 (1.0–2.0)	*Z* = −2.803 *p* = 0.005^*^d = 0.974
Increased motivation and ability of thinking	2.7 ± 0.7	3.0 (2.5–3.0)	2.3 ± 0.8	2.0 (2.0–3.0)	*Z* = −2.833 *p* = 0.005^*^d = 0.987
Active class atmosphere	2.6 ± 0.8	3.0 (2.0–3.0)	2.5 ± 1.0	2.0 (2.0–3.0)	*Z* = −0.646 *p* = 0.518 *d* = 0.203
Promoted teamwork spirit and ability	2.6 ± 0.9	3.0 (2.0–3.0)	2.3 ± 1.0	2.0 (2.0–3.0)	*Z* = −1.436 *p* = 0.151 *d* = 0.460
Developed clinical problem-solving skills	3.0 ± 0.7	3.0 (3.0–3.0)	2.9 ± 0.8	3.0 (2.0–3.0)	*Z* = −0.845 *p* = 0.398 *d* = 0.266
Improved understanding of important and difficult topics	3.2 ± 0.6	3.0 (3.0–4.0)	2.8 ± 0.6	3.0 (2.0–3.0)	*Z* = −2.433 *p* = 0.015^*^d = 0.822
**Perceptions and evaluation of the BL intervention (1–10 points)**					
Course content	7.3 ± 1.8	8.0 (6.0–8.0)	6.7 ± 1.8	7.0 (6.0–8.0)	*Z* = −1.920 *p* = 0.055 *d* = 0.646
Course organization	7.4 ± 2.1	8.0 (7.0–9.0)	6.5 ± 1.9	6.0 (5.0–8.0)	*Z* = −2.389 *p* = 0.017^*^d = 0.828
Educational material	7.2 ± 2.0	7.0 (6.0–9.0)	6.1 ± 1.8	6.0 (5.0–7.0)	*Z* = −3.283 *p* = 0.001^*^d = 1.236
Easiness to understand	7.1 ± 2.0	7.0 (6.0–9.0)	6.3 ± 1.5	6.0 (5.0–7.0)	*Z* = −2.698 *p* = 0.007^*^d = 0.958
Comprehensive coverage of the subject	7.4 ± 1.7	8.0 (7.0–8.0)	6.4 ± 1.5	6.0 (5.0–7.0)	*Z* = −3.249 *p* = 0.001^*^d = 1.218
Clarity of instructions	7.4 ± 2.0	8.0 (7.0–9.0)	6.5 ± 1.6	6.0 (5.0–7.0)	*Z* = −2.85 *p* = 0.004^*^d = 1.026
Motivating study timetable	7.1 ± 2.0	7.0 (5.0–9.0)	7.0 ± 1.5	7.0 (6.0–8.0)	*Z* = −0.352 *p* = 0.725 *d* = 0.113
Self-assessment test	7.0 ± 1.6	7.0 (6.0–8.0)	7.2 ± 1.6	7.0 (6.0–8.0)	*Z* = −0.559 *p* = 0.576 *d* = 0.180

Data are expressed as mean ± standard deviation and median (interquartile range). BLG, blended-learning group; FLG, face-to-face learning group. ^*^Significant differences between groups.

Table 4 shows the results for usability and BL acceptance of the BLG at the end of the study. In relation to the usability of the platform, the SUS total score was 76.9 ± 15.2 out of 100 points, thus acceptable usability was reported. In addition, the BL acceptance showed that the values of perceived easiness of use and usefulness were higher than 6 over 7 points, whilst attitude toward use and behavioral intention to use had values > 5.5 over 7.

**Table 4:** Results for usability and blended learning acceptance in the blended learning group after the intervention.

	**BLG (*****n*** = **48)**	
	**Mean** ±**SD**	**Median (IQR)**
**System usability score (1–5 points)**		
I think that I would like to use this platform frequently	4.0 ± 0.8	4.0 (3.0–5.0)
I found the platform unnecessarily complex	1.9 ± 0.8	2.0 (1.0–2.0)
I thought the platform was easy to use	4.2 ± 0.9	4.0 (4.0–5.0)
I think that I would need the support of a technical person to be able to use this platform	1.6 ± 1.0	1.0 (1.0–2.0)
I found the various functions in this platform were well-integrated	4.0 ± 0.8	4.0 (4.0–4.0)
I thought there was too much inconsistency in this platform	2.2 ± 1.0	2.0 (2.0–3.0)
I would imagine that most people would learn to use this platform very quickly	4.2 ± 0.8	4.0 (4.0–5.0)
I found the platform very cumbersome to use	1.7 ± 0.8	2.0 (1.0–2.0)
I felt very confident using the platform	3.7 ± 1.0	4.0 (3.0–4.0)
I needed to learn a lot of things before I could get going with this platform	1.8 ± 0.9	2.0 (1.0–2.0)
Total score (max 100 points)	76.9 ± 15.2	77.5 (67.5–87.5)
**BL acceptance (1–7 points)**		
Perceived easiness of use	6.5 ± 0.8	7.0 (6.0–7.0)
Perceived usefulness	6.2 ± 0.9	6.0 (6.0–7.0)
Behavioral intention to use	5.7 ± 1.2	6.0 (5.0–7.0)
Attitude toward use	5.8 ± 1.2	6.0 (5.0–7.0)

Data shown as mean ± standard deviation and median (interquartile range). BLG, blended learning group.

## Discussion

Our findings demonstrate that the BL teaching modality produced an improvement in knowledge, ethical and gender competencies, satisfaction, motivation, thinking skills, comprehension, and organization, and thus obtained better scores than the FL.

These results are in the line with those reported by Ødegaard et al. (12) who concluded that the use of interactive websites/apps combined with face-to-face methodologies in physiotherapy education is as effective as traditional methods when assessing knowledge acquisition, practical skills, and the students' perceptions. In our study, in terms of knowledge, the BLG group acquired more theoretical and practical knowledge than the FLG. This may be due to the fact that students are more involved in their learning and acquire an active role by carrying out online activities (46), rather than just passively listening to the lessons. In addition, the content is reinforced when it is explained by the professor (27). Moreover, the BL group also received digital lectures that could be attended at home, while active learning activities were used in the classroom. In this regard, it should be taken into account that reverse classroom teaching leads to a learning environment that aims to develop higher order cognitive skills (47). It has been shown that flipped classroom teaching has the potential to enhance the higher-order thinking skills and self-regulated learning among students (48–50). Our findings are in line with the study carried out by Arroyo-Morales et al. (51) who suggested that a BL intervention including free access to a specific website on human anatomy improved knowledge acquisition regarding knee palpation and ultrasound skills compared to just receiving printed information. In addition, our students could access more visual and interactive content while also being able to consult the information as many times as needed which could justify these results. Moreover, the latter study performed by Arroyo-Morales et al. showed that satisfaction levels were high in the BLG (4.1 out of five points), which is in accordance with the present study (three out of four points).

In the same vein, we could affirm that BL showed good usability and acceptance since most of the students in our study would like to use the online platform frequently, and considered it to be easy to use with well-integrated functions, and they felt confident when using the platform. Similarly, Lozano-Lozano et al. (52) described significant improvements in motivation, mood state, and satisfaction when using an interactive platform accessible through mobile devices and ultrasound imaging compared to traditional teaching in physiotherapy students. This is of great importance as motivation is an essential factor in the learning process (53). Moreover, satisfaction with the use of an online adaptive e-learning platform and assessment software that promotes critical thinking in medical students in a cardiovascular system course was recently studied. In this regard, not only the students were satisfied with this methodology, but the use of the platform correlated with higher performance on course exams (54).

The effectiveness of the BL method has been previously demonstrated in other health science degrees. For example, our findings support the results of Makhdoom et al. (17) who explored the effectiveness of BL in medicine education. They showed that a BL modality was significantly better than traditional learning in all domains of the educational context except for the social aspect, as well as in all types of exams (written, practical, and case resolution). These findings suggest that healthcare students are keen to new learning methods and BL may be an effective educational approach. However, Ilic et al. (55) conducted a study with 147 medical students that were randomly assigned to receive either a BL intervention or a didactic learning approach. Both approaches were effective at increasing the knowledge and skills of the students, although BL was significantly more effective at increasing the attitudes toward evidence-based medicine and its self-reported use in clinical practice. In contrast, in the study performed by Atwa et al., the sampled medical students and faculty members preferred FL and considered BL to be an acceptable and practical solution for the post-COVID-19 era (56).

For all these reasons, and given how currently BL seems more to be than a proposal in reality, it seems necessary to adapt the university environment and to develop the digital competences of the teachers and students (57). This is one of the challenges that Higher Education should address.

Although other investigations have reported the benefits of BL in specific aspects such as technology usage, interactions between students, the tutoring model, feedback, and student support (57), it seems to be difficult to compare these results with those of other trials due to the different teaching methodologies that usually take place under the name of BL and the different questionnaires and scales employed to measure the results. Therefore, the development of a BL structured protocol is needed to perform future studies along the same lines.

### Limitations and strengths

This study presents both strengths and limitations. Prior to this study, there was scarce information about BL interventions related to future cardiac physiotherapy professionals. In addition, there is a scarcity of randomized trials using BL among physiotherapy students. Moreover, this study highlights the inclusion of several online resources based in scientific evidence in the BL intervention that are not usually included or detailed in other studies. Nevertheless, our results must be taken with caution because of the short period evaluated, thus we recommend that future research investigate the long-term effects of BL. Moreover, since the research sample consisted of physiotherapy students from a single university in Spain, it is difficult to generalize the results to other university degrees and may have low external validity. It should be taken into account that several non-validated instruments were used. In addition, some of the analyzed outcomes are the self-perceptions of the study participants, therefore further studies that use objective measurements are highly recommended in this regard. Exploring the effects on the psychomotor skills of students would be an interesting aspect to evaluate when performing this type of study. It would be interesting for future research to mimic this work in other university degrees to obtain more representative results. Future studies should consider these limitations and investigate how digital learning designs could facilitate the students' learning, practical skills, and behaviors, as well as determine the useful benchmarks as part of improving the teaching-learning process in digital educational environment combined with face-to-face methodologies.

## Conclusions

The BL intervention can be used for improving knowledge acquisition, ethical and gender competencies, the motivation to prepare students before class, motivation and the ability of thinking, and the understanding of important topics. In addition, the participants highlighted the organization of the course, the educational material provided, the easiness of understanding the subject, the comprehensive coverage of the subject, as well as the clarity of the instructions. Furthermore, the level of BL acceptance was positive and its usability was determined to be acceptable. Further studies are needed to confirm our findings in a larger population.

## Recommendations

According to the results of the present study, the implementation of BL in physiotherapy and in health professional education is recommended to improve the knowledge, competencies, perceptions, and satisfaction of students. Overall, this study supports the use of BL as a pedagogical approach to foster innovative learning.

## Data availability statement

The original contributions presented in the study are included in the article/supplementary material, further inquiries can be directed to the corresponding author.

## Ethics statement

The studies involving human participants were reviewed and approved by the Ethics Committee of the University of Valencia, Spain (protocol registration number: IE1576047). The participants provided their written informed consent to participate in this study.

## Author contributions

EM-S conceived and designed the study. EM-S, NC-S, and TS-M conducted the intervention and evaluation sessions and entered the data. JC and NC-S analyzed the data. EM-G, NM-S, SP-A, NC-S, and JS-G drafted the manuscript. RJ-V and RR supervised the manuscript. All authors reviewed and approved the final version of the paper.
